# A randomised controlled trial to examine the effects of cinacalcet on bone and cardiovascular parameters in haemodialysis patients with advanced secondary hyperparathyroidism

**DOI:** 10.1186/s12882-021-02312-2

**Published:** 2021-03-23

**Authors:** Helen Eddington, Rajkumar Chinnadurai, Helen Alderson, Sara T. Ibrahim, Constantina Chrysochou, Darren Green, Ibi Erekosima, Alastair Hutchison, Abdalla Bubtana, Janet Hegarty, Philip A. Kalra

**Affiliations:** 1 Renal Department, University Hospitals of Birmingham NHS Trust, Birmingham, UK; 2 Department of Renal Medicine, Salford Royal NHS Foundation Trust, Salford, M6 8HD UK; 3 Faculty of Biology, Medicine and Health, University of Manchester, Manchester, UK; 4 Department of Internal Medicine and Nephrology, Faculty of Medicine, Alexandria University, Alexandria, Egypt; 5 Dorset County Hospital Foundation Trust, Dorset, UK; 6 Department of Renal Medicine, Manchester University NHS Foundation Trust, Manchester, UK

**Keywords:** Chronic kidney disease-mineral bone disorder (CKD-MBD), Cinacalcet, Secondary hyperparathyroidism, Left ventricular mass index (LVMI), Parathormone (PTH)

## Abstract

**Background:**

Secondary hyperparathyroidism may lead to increased cardiovascular risk. The use of cinacalcet may improve bone and cardiovascular health with improved parathormone (PTH) and phosphate control.

**Methods:**

This is an open-label prospective randomised controlled trial to compare progression of cardiovascular and chronic kidney disease mineral and bone disorder (CKD-MBD) parameters. Patients were randomised to receive cinacalcet alongside standard therapy or standard therapy alone. Thirty-six haemodialysis patients who had > 90 days on dialysis, iPTH > 300 pg/mL, calcium > 2.1 mmol/L and age 18–75 years were included. Following randomization, all 36 patients underwent an intensive 12-week period of bone disease management aiming for iPTH 150-300 pg/mL. The primary outcome was change in vascular calcification using CT agatston score. Secondary outcomes included pulse wave velocity (PWV), left ventricular mass index (LVMI), carotid intima-media thickness (CIMT), augmentation index (Aix) and bone measurements. The above measurements were obtained at baseline and 12 months.

**Results:**

There was no evidence of a group difference in the progression of calcification (median change (IQR) cinacalcet: 488 (0 to1539); standard therapy: 563 (50 to 1214)). In a post hoc analysis combining groups there was a mean (SD) phosphate reduction of 0.3 mmol/L (0.7) and median (IQR) iPTH reduction of 380 pg/mL (− 754, 120). Regression of LVMI and CIMT was seen (*P =* 0.03 and *P =* 0.001*)* and was significantly associated with change of phosphate on multi-factorial analyses.

**Conclusions:**

With a policy of intense CKD-MBD parameter control, no significant benefit in bone and cardiovascular markers was seen with the addition of cinacalcet to standard therapy over one year. Tight control of hyperphosphataemia and secondary hyperparathyroidism may lead to a reduction in LVMI and CIMT but this needs further investigation**.** Although the sample size was small, meticulous trial supervision resulted in very few protocol deviations with therapy.

**Supplementary Information:**

The online version contains supplementary material available at 10.1186/s12882-021-02312-2.

## Background

Cardiovascular disease is the leading cause of death in patients with chronic kidney disease (CKD) [1]. Studies have shown that derangements of intact parathyroid hormone (iPTH), phosphate and calcium are linked to increased mortality [2, 3]. A higher iPTH has also been associated with increased calcification and cardiomyopathy [4, 5]. Cinacalcet enabled increasing numbers of patients to achieve targets for CKD mineral and bone disorder (CKD-MBD), although there is continued restriction on its use [6, 7]. Since the start of this trial there has been some evidence that calcimimetics may reduce progression of vascular calcification [8–10]. Calcification progression has been studied with cinacalcet and other CKD-MBD medications but the few randomised trials have allowed a disparity of parathyroid hormone or phosphate control between arms so that uncertainty has remained regarding whether it is the medication or the control that has been beneficial [11–13].

The primary aim of this study was to determine if cinacalcet treatment and standard therapy attenuated progression of vascular calcification compared to standard therapy in haemodialysis patients when equivalent control of secondary hyperparathyroidism was achieved between treatment arms. Our trial is different from the ADVANCE trial in that we aimed for equivalent biochemical targets in the treatment arms and did not specify a maximum vitamin D dose [14].

The KDIGO guidelines published in 2009 suggest aiming for an iPTH level within the range of 2–9 times the upper limit of normal [15]. A subsequent observational study by Floege et al. showed a survival benefit in patients achieving the tighter KDOQI iPTH target of 150-300 pg/mL along with the targets for calcium (2.10–2.37 mmol/L) and phosphate (1.13–1.78 mmol/L) [16, 17]. In light of this we performed a post-hoc analysis investigating the whole trial population to determine if any differences in endpoints were associated with changes in PTH, phosphate and other biomarkers.

## Materials and methods

This open label randomized control trial compared cinacalcet versus standard therapy in patients with secondary hyperparathyroidism and dialysis dependent end stage kidney disease to examine the effect of cinacalcet on bone and cardiovascular parameters. All patients gave written informed consent to participate in the trial. Ethical and MHRA approval was granted by Salford and Trafford Local Research Ethics Committee, approved in November 2005 (ref: 05/Q1404/216). The trial conformed to the standards outlined in the 1974 Declaration of Helsinki and was registered with the ISRCTN registry (ISRCTN81718275) (31/07/2008).

Patients with secondary hyperparathyroidism who had been receiving dialysis for greater than 90 days were screened for enrolment into the study. Inclusion criteria included an iPTH > 300 pg/mL, corrected calcium > 2.1 mmol/L and age 18-75 years. Exclusion criteria included previous coronary stents, coronary artery bypass grafts or valve replacement, atrial fibrillation and severe liver disease.

The randomisation was stratified by diabetic status and baseline pulse wave velocity. Following randomisation all patients underwent a 12-week intensive management phase with both medical intervention and dietary advice. During this calcium and phosphate concentrations were measured fortnightly and the iPTH was measured at weeks 2 and 12. Unrestricted phosphate binders (i.e. calcium and non-calcium containing preparations) and flexible doses of vitamin D sterols were available for use in all patients. The targets for treatment (range of phosphate, corrected calcium and iPTH) and the strategies (dose adjustment of cinacalcet, one-alfacalcidol and phosphate binders) followed to achieve these targets are included in the supplementary file 1.

Following the intensive management phase, patients were reviewed every 8 weeks until the final visit at week 52 unless a medication change or biochemical result warranted more frequent review. Biochemical and haematology tests were measured at each time-point. All patients received a minimum of 12 consultations throughout the trial.

**Fig. 1:**
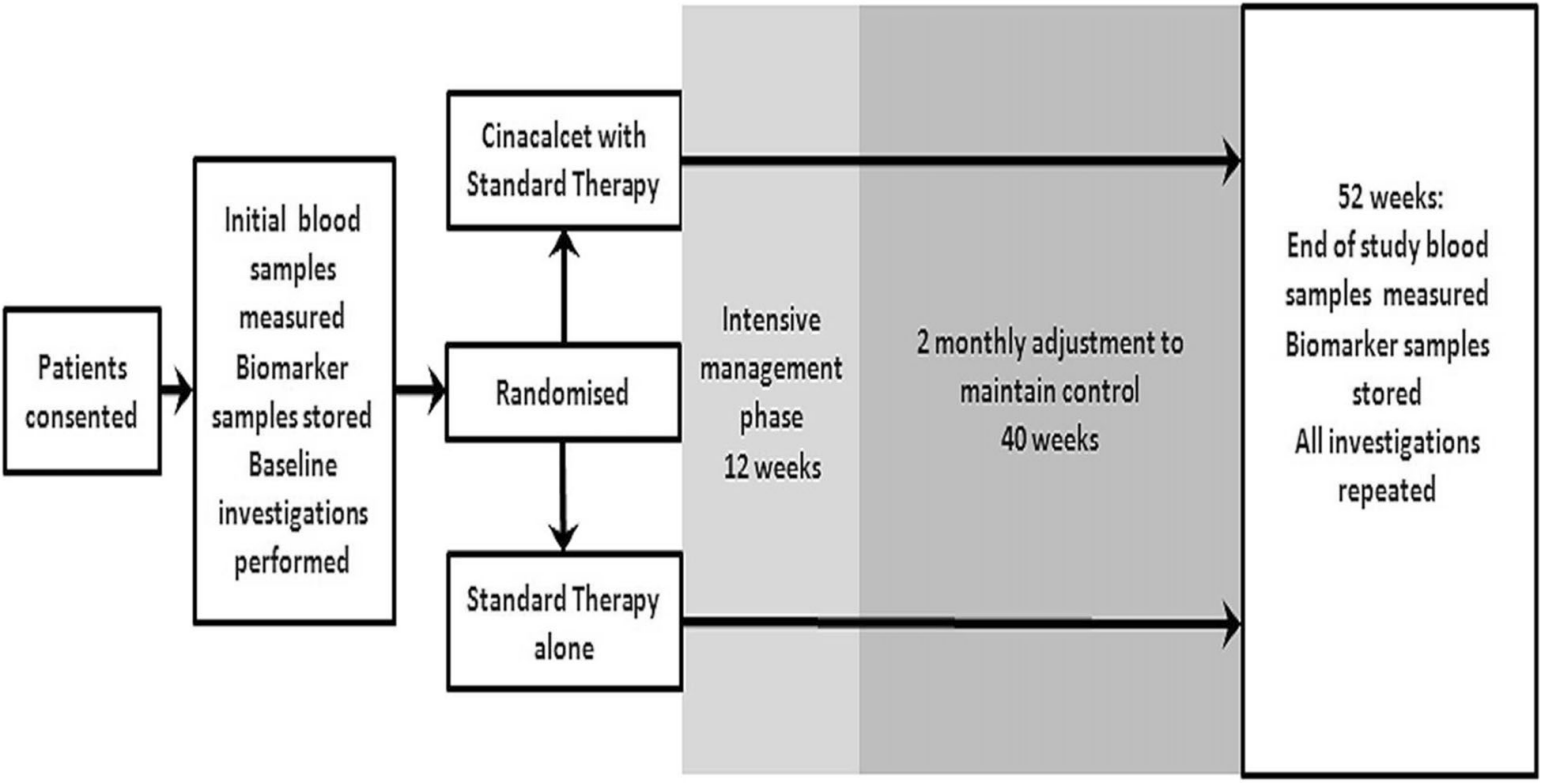
Study protocol

The study protocol is outlined in Fig. 1.

The following investigations were performed at baseline and at 12 months; serum and plasma samples were also taken and stored at these time points.

Scans were performed on the same post-dialysis day at baseline and 12 months and were interpreted by clinicians blinded to randomisation and blood results:

- Cardiac and abdominal aorta vascular calcification score (Agaston method, GE CT Lightspeed 16-slice scanner).
- Cardiac structure and function including left ventricular mass index (LVMI). (Cardiac magnetic resonance (CMR) scan).
- Bone mineral density measurements included QCT spine (GE scanner using Mindways software), Dual X-Ray Absorptiometry (DXA) of Hip and spine and peripheral QCT (pQCT) of forearm.

The following bedside investigations were performed immediately after dialysis with the patient at their dry weight as determined by their medical team:

- Carotid-femoral pulse wave velocity (cfPWV) and augmentation index (Aix) at the radial artery (SphygmoCor® (Atcor Medical)).
- The carotid-intima media thickness (CIMT) was measured 1 cm below the bifurcation of the carotid artery bilaterally.

Adequate dialysis was determined as a Kt/v > 1.2 and / or a URR > 65%.

Biochemical and haematology pre-dialysis samples were processed at 12 time points throughout the trial. All biochemical samples were analysed in a central laboratory (Roche modular analyser except iPTH (DPC immulite 2000 chemiluminescent immunoassay)). Haematology results were analysed using Sysmex XE-1200. The biomarkers were analysed as following. FGF-23:Immunotiopics Inc. 2nd generation ELISA (San Clemente, CA, USA); 25(OH) vitamin D: HPLC tandem mass spectrometry; 1,25(OH)_2_ vitamin D: RIA (Immuno Diagnostic Systems (IDS), Boldon, UK); NTProBNP and Troponin T: ECLIA Modular Analytics E170 analyser (Roche Diagnostics, Lewes, UK); Fetuin A: ELISA (BioVendor GmbH, Heidelberg, Germany); Osteoprotegerin: ELISA (IDS, Boldon, UK).

The primary outcome measure for the trial was change in calcification score at 12 months. The secondary outcomes were change in arterial stiffness, bone mineral density, cardiac morphology, survival and biomarkers.

At the time of designing this study there were minimal data available upon which to base a potential effect size for the primary endpoint. The trial was designed to have an 80% power to detect a one standard deviation difference in absolute change in calcification score over 1 year at the 5% significance level. A power calculation determined that 32 patients would be required for the study to have adequate power; the recruitment target was set at 40 and 36 patients were randomized.

### Statistical analysis

Categorical data were analysed using chi-square analysis. Continuous data were assessed for normality using graphical means and transformed as necessary. All data were then analysed using linear regression adjusted for diabetes and PWV as per randomisation stratification. Differences over 12 months were assessed by Wilcoxon signed-rank test or paired t-test depending on normality of data. Multi-factorial linear regression was used to determine further associations using normalised data where required. The study end points for the long term follow up included the first of the following: death, renal transplant, last follow up date or end of analysis period, which was 31st December 2018. Survival analyses was performed using cox regression analyses to study the difference in mortality between the two groups (cinacalcet and control). A post-hoc analysis to examine the overall effects of achieving tight iPTH control was also undertaken, which aggregated the data of the 36 enrolled patients. To overcome the limitation of a smaller sample size, we conducted an inverse probability of treatment weighting (IPTW) analysis by propensity scores to generate a balanced weighted sample [18]. The propensity scores were generated by binary logistic regression incorporating all the baseline study variables without any missing values. Further linear regression analysis was conducted using this weighted sample which had been generated to calculate the estimated effect of intervention on the difference in the outcome measures between baseline and follow-up. All statistical analyses were performed using SPSS version 22.0 registered to the University of Manchester.

## Results

**Fig. 2:**
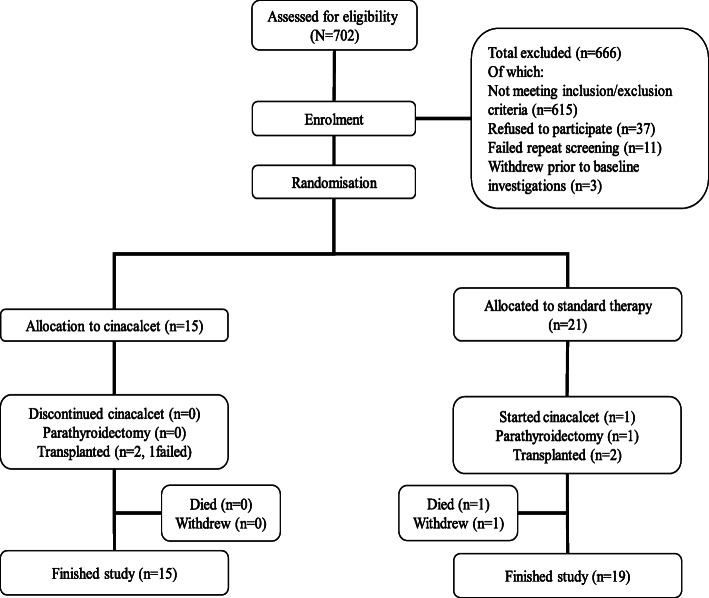
A consort diagram detailing the recruitment and trial completion

Figure 2 shows a consort diagram detailing the recruitment and trial completion. The baseline characteristics of the enrolled population are shown in Table 1 and the baseline cardiovascular and bone imaging investigations are shown in Table 2. Four patients did not undergo CMR investigations due to claustrophobia but continued in the study. The mean pulse wave velocity (8.6 (3) m/s), total calcification score (2174 (0 to 16,635) and left ventricular mass index (109 (39) g/m^2^) were high at baseline as would be expected in a haemodialysis population.

**Table 1:** Baseline demographics, overall and per treatment arm

	All patients (*n* = 36) Mean (SD) / Median [range]/ *n* (%)	Cinacalcet (*n* = 15) Mean (SD) / Median [range] / *n* (%)	Control (*n* = 21) Mean (SD) / Median [range]/ *n* (%)	*p*-Value
Demographics				
Age	51 (15)	45 (16)	54 (13)	0.068
Male	23 (64%)	11 (73%)	12 (57%)	0.319
Caucasian	30 (83%)	14 (93%)	16 (76%)	0.174
Diabetes	4 (11%)	1 (7%)	3 (14%)	0.473
Smoking	9 (25%)	5 (33%)	4 (19%)	0.329
Height (cm)	166 (11)	169 (11)	163 (11)	0.298
Weight (kg)	73 (15)	74 (15)	72 (15)	0.626
BMI	26 (5)	25 (5)	26 (5)	0.927
Cardiovascular				
SBP (mmHg)	131 (26)	133 (27)	130 (26)	0.789
DBP (mmHg)	72 (13)	72 (13)	72 (13)	0.877
MI	5 (14%)	2 (13%)	3 (14%)	0.935
Angina	7 (19%)	5 (33%)	2 (10%)	0.075
Stroke	3 (8%)	0	3 (14%)	0.126
TIA	7 (19%)	2 (13%)	5 (24%)	0.433
All CVD	14 (39%)	6 (40%)	8 (38%)	0.908
NYHA score	1: 26 (72%)	1: 10 (67%)	1: 16 (76%)	0.529
	2–3:10 (28%)	2–3:5 (33%)	2–3:5 (24%)	
CCS score	0:33 (92%)	0: 14 (93%)	0: 19 (91%)	0.759
	1–3: 3 (8%)	1–3: 1 (7%)	1–3: 2 (9%)	
Renal history				
Adequate dialysis	23 (80%)	8 (73%)	15 (83%)	0.265
Dialysis duration (m)	38 [6–319]	33 [6–319]	43 [10–222]	0.072
Previous transplant	9 (25%)	4 (27%)	5 (24%)	0.845
Hyperparathyroidism medication at baseline				
Calcium binder	15 (42%)	7 (47%)	8 (38%)	0.607
Non-calcium binder	30 (83%)	13 (87%)	17 (81%)	0.650
VDRA	35 (97%)	15 (100%)	20 (95%)	1.000

*MI* myocardial infarction, *TIA* transient ischaemic attack, *CVD* cardiovascular disease, *NYHA* New York Heart Association heart failure score, *CCA* Canadian Cardiovascular Society angina score, *VDRA* vitamin D receptor analogue, *iPTH* intact parathyroid hormone, *CRP* C-reactive protein, *SBP* systolic blood pressure, *DBP* diastolic blood pressure, *BMI* Body mass index

**Table 2:** Baseline and follow-up investigations overall and per treatment arm

Mean (SD) / Median [range] / *n* (%)	All patients (*n* = 36)	Cinacalcet (*n* = 15)		Control (n = 21)		Estimated effect (CI)	*p*-Value
		Baseline	12 months	Baseline	12 months		
iPTH (pg/mL)	730 [318 to 1586]	665 [353 to1586]	225 [152 to 386]	806 [318 to 1096]	294 [145 to 445]	146 (− 240, 355)	0.7
Calcium (mmol/L)	2.30 (0.14)	2.32 (0.16)	2.34 (0.17)	2.29 (0.12)	2.39 (0.24)	0.08 (−0.3,0.03)	0.2
Phosphate (mmol/L)	1.91 (0.58)	1.91 (0.58)	1.62 (0.56)	1.91 (0.59)	1.61 (0.65)	0.2 (− 0.3,0.7)	0.5
Haemoglobin (g/L)	124 (15)	124 (18)	119 (20)	124 (14)	119 (13)	7.8 (−17, 36)	0.5
CRP	11 [0,97]	16 [0 to 97]	4 [0.6 to 17]	7 [0.5 to 52]	5 [1.6 to 7.4]	5.1 (−13.1, 8)	0.6
Medication							
Calcium Binder	15 (42%)	7 (47%)	8 (53%)	8 (38%)	7 (35%)		0.3
Non-calcium binder	30 (83%)	13 (87%)	12 (80%)	17 (81%)	12 (60%)		0.3
VDRA	35 (97%)	15 (100%)	14 (93%)	20 (95%)	20 (91%)		0.4
Vascular stiffness & Carotid intima media thickness							
cfPWV (m/s)	8.6 (3)	7.8 (2)	8 (2)	9.2 (3)	9.5 (2.6)	−0.2 (−1.6,1.2)	0.8
Augmentation index (%)	21 [−10,+ 53]	21 [−5 to + 37]	21 [10 to 26]	26 [− 10 to + 53]	28 [16 to 35]	6 (−21, 4)	0.2
Average CIMT (mm)	0.04 (0.01)	0.04 (0.01)	0.03 (0.01)	0.04 (0.01)	0.04 (0.01)	−0.004 (− 0.1,0.002)	0.2
Agatston calcification score							
Coronary calc score	204 [0 to 4075]	96 [0 to 4075]	106 [18 to 950]	260 [8 to 3785]	464 [60 to1480]	218 (− 502,387)	0.8
Aortic calc score	892 [0 to 16,466]	302 [0 to 13,063]	480 [60 to 2224]	2090 [0 to 16,466]	2665 [895 to 8567]	393 (− 382,1224)	0.3
Total calc score	2174 [0 to 16,635]	814 [0 to 15,090]	852 [165 to 5942]	3401 [7.5 to 16,635]	4768 [996 to 9582]	455 (− 564,1293)	0.4
Cardiac magnetic resonance	(*n* = 32)	(*n* = 12)		(*n* = 20)			
LV mass index (g/m^2^)	109 (39)	120 (43)	92 (31)	103 (37)	90 (20)	−0.2 (−1.2,0.8)	0.7
LV stroke volume (mL)	92 (22)	102 (22)	103 (24)	87 (19)	98 (22)	−0.5 (−16,15)	0.9
LV ejection fraction (%)	68 (11)	70 (7)	70 (8)	67 (13)	70 (10)	−0.5 (−7,6)	0.9
Bone density	(*n* = 36)	(*n* = 15)		(*n* = 19)			
DXA spine BMD g/m2	1.1 (0.2)	1.1 (0.1)	1.1 (0.1)	1.1 (0.2)	1.1 (0.2)	−0.03 (−0.07,0.001)	0.1
DXA hip BMD	0.9 (0.1)	0.9 (0.1)	0.9 (0.1)	0.8 (0.2)	0.8 (0.2)	−0.01 (−0.03,0.2)	0.4
DXA femoral BMD	0.8 (0.1)	0.9 (0.1)	0.9 (0.1)	0.8 (0.1)	0.8 (0.2)	−0.01 (− 0.04,0.02)	0.6
QCT BMD	126 (48)	134 (38)	136 (38)	120 (54)	120 (40)	−2.7 (−14,9.1)	0.6
pQCT50 cortcnt	80 (30)	86 (23)	90 (21)	75 (33)	82 (27)	5.1 (−2.5,12.8)	0.2
Stress strain index	215 (99)	227 (68)	244 (66)	207 (54)	202 (94)	28 (−3.8,59.6)	0.08
Biomarkers							
NTproBNP (pg/mL)	2945[332to161680]	2483[679to80840]	3066 [90to49520]	3011[332to161680]	3208 [107to126250]	0.01 (−0.4,0.4)	0.9
Troponin T (ug/L)	33 [11 to 199]	27 [11 to 89]	30 [4 to 1478]	38 [12 to 198]	42 [14 to 131]	0.1 (−0.2,0.4)	0.5
25(OH) Vitamin D (nmol/L)	13 [4 to 33]	16 [6 to 59]	16 [4 to 34]	11 [4 to 33]	13 [4 to 33]	−0.1 (−0.3,0.7)	0.2
1,25 (OH)_2_ Vitamin D (pmol/L)	66 [34 to 132]	62 [34 to 130]	65 [50 to 91]	70 [39 to 132]	78 [54 to 122]	−0.1 (−0.3,0.8)	0.3
Osteoprotegerin (pmol/L)	8 [3 to 23]	8 [3 to 17]	8 [3 to 18]	8 [4 to 23]	8 [4 to 17]	0.4 (−0.4,0.1)	0.3
FGF23 (RU/mL)	13,794 [412 to 149,994]	22,243 [774 to 149,994]	7422 [328 to 117,011]	13,793 [412 to 60,933]	11,540 [195 to 143,782]	−0.06 (−0.6,0.5)	0.8
Fetuin A (ng/mL)	342 [190 to 588]	363 [290 to 562]	369 [154 to 538]	321 [190 to 588]	343 [161 to 580]	−0.2 (−0.9,0.6)	0.7

*iPTH* intact PTH, *VDRA* vitamin D receptor analogues, *cfPWV* carotid-femoral pulse wave velocity, *CIMT* carotid intima-media thickness, *calc* calcification, *LV* left ventricular, *DXA* Dual X-Ray Absorptiometry, *BMD* bone mineral density, *QCT* quantitative computerised tomography, *pQCT50* peripheral QCT at 50% of forearm, *crtcnt* cortical content, *NTproBNP* N-Terminal Pro Brain Naturetic peptide, *FGF23* fibroblast growth factor 23

There were no substantive differences in most baseline parameters between treatment arms except that the baseline median calcification score, iPTH and mean cfPWV were higher in the standard therapy arm (cinacalcet arm: 814, 665 pg/ml, 7.8 (2) compared to 3401, 806 pg/ml, 9.2 (3) respectively). Fifteen patients were randomised to cinacalcet with standard therapy compared to 21 with standard therapy alone: the difference due to chance imbalance of strata at randomisation. Two patients in the standard therapy arm did not complete the study; one died and the other withdrew in week 51.

Adverse events were similar in both arms during the trial (Table 3). There was a higher incidence of hypocalcaemia in patients randomised to cinacalcet compared to standard therapy as would be expected. No patients discontinued cinacalcet in the study.

**Table 3:** Overview of all adverse events reported during the trial (1 year follow up)

Description	Cinacalcet No of patients (no of events)	Control No of patients (no of events)	Details of events	*p*-Value
Infection	2 (3)	6 (7)		0.43
Renal transplant	1	2 (2)		0.7
Vascular access procedures	4 (11)	1		0.06
Death		1	brain tumour	0.4
Parathyroidectomy		1		0.4
Low calcium	11 (20)	4 (5)	< 2.1 mmol/L	0.01
High calcium	2 (5)	9 (17)	> 2.6 mmol/L	0.06
Claustrophobia	3	1	In MR scan	0.4
Other	11 (16)	12 (20)		0.4

### Primary endpoint

**Fig. 3:**
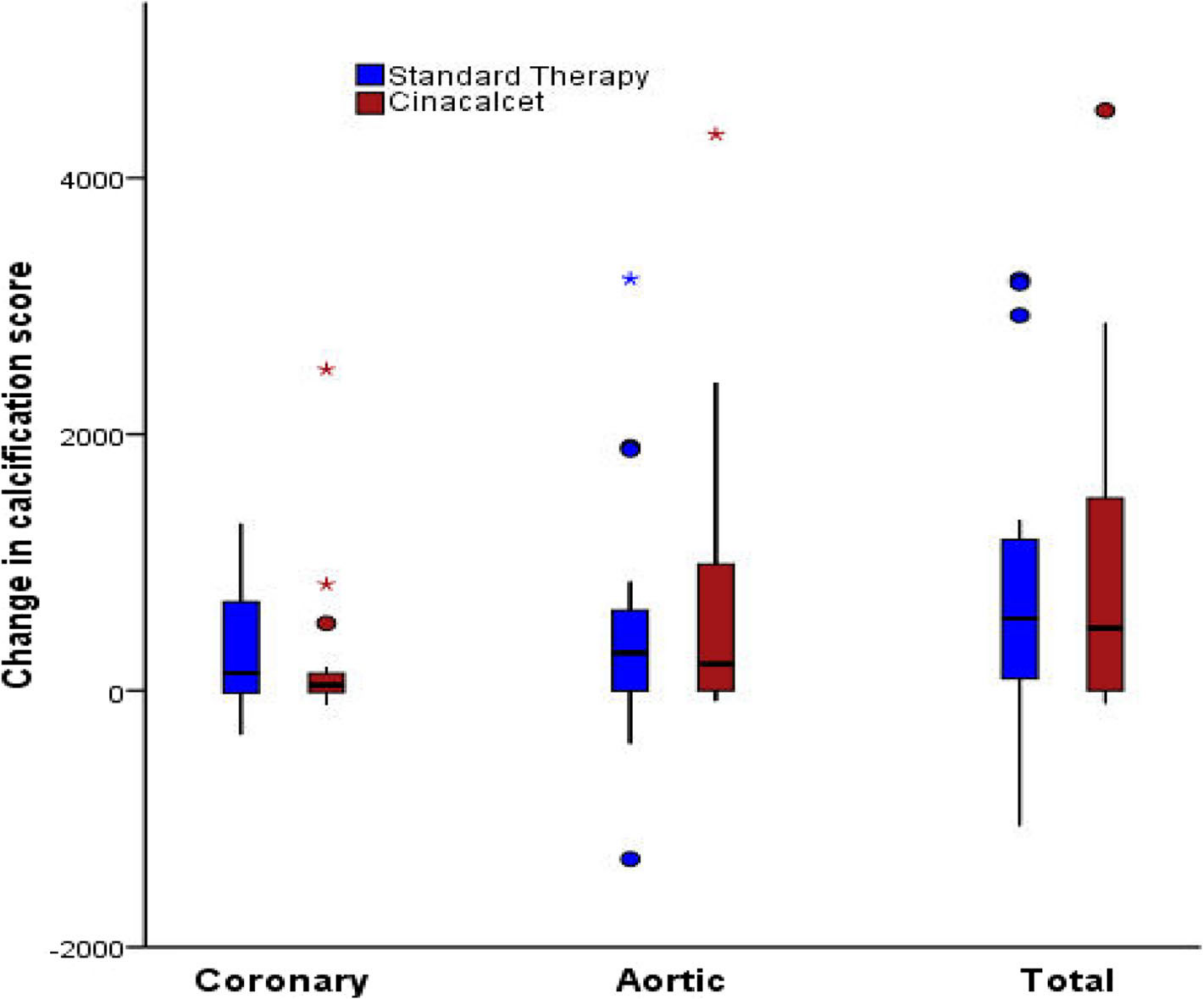
Change of calcification score using the Agatston method. No significant difference is seen between treatment arms

The primary outcome was absolute change of total calcification score from baseline to 1 year (Fig. 3). Although there was no significant difference between the study arms (median change cinacalcet 488; standard 563: Estimated effect (EE) = 5.0 (− 0.1–10, *P* = 0.053) changes were in a direction that favoured cinacalcet. When areas of calcification were analysed separately no significant change was seen with either coronary (median change cinacalcet 43; standard 207: EE = -0.9(− 5.9, 4.1 *P =* 0.7) or aortic calcification (median change cinacalcet 207; standard 293: EE =4.6(− 0.5, 9.7 *P =* 0.08). Four patients who completed the trial had a total calcification score < 30 at baseline; three of which still had a calcification score < 30 at 12 months. The median percentage progression of total calcification in all study patients was 20% (IQR 0, 72%) (coronary 11% (IQR 28, 52%); aortic 12% (IQR 0, 63%)). The IPTW method generated a weighted sample as demonstrated in the supplementary file 2. The estimated effect of the outcomes calculated from the weighted sample was not significantly different between the study arms (supplementary file 3).

### Secondary endpoints

Similar iPTH control was seen in both study arms (median iPTH at 12 months (pg/ml): cinacalcet 225 (152,386); standard 294 (145,445)). The area under the curve for PTH exposure was calculated with no significant difference found between the groups (*P* = 0.3). Similarly, no significant difference was seen with exposure to phosphate over the 12-month period with mean phosphate at 12 months in cinacalcet group 1.62 mmol/L (0.56) and 1.62 mmol/L (0.65) with standard therapy (*P* = 0.6). There was no significant difference in change or final vitamin D dose, calcium binder dose or calcium dialysate between groups. Twelve patients in each treatment arm were prescribed a non-calcium phosphate binder (*P =* 0.3).

**Fig. 4:**
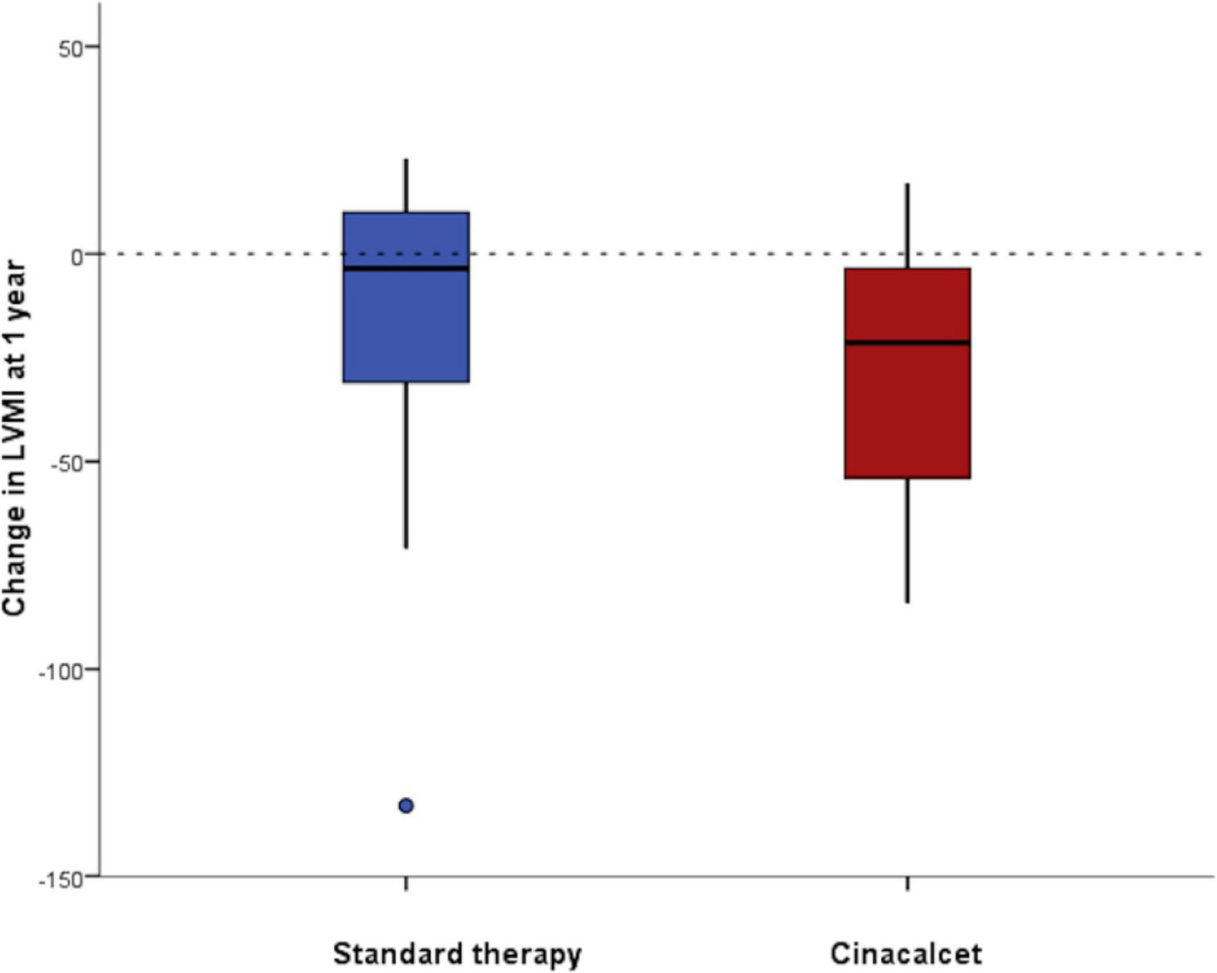
Change in LVMI between the treatment arms at one year

The cfPWV and radial Aix increased during the study despite improved control of secondary hyperparathyroidism. The progression was lower in the cinacalcet arm though this was not significant (Table 2). Despite the increase in calcification and vascular stiffness over the study period there was a reduction in LVMI in both treatment arms (Fig. 4). The difference between arms was not statistically significant. No significant change was seen for other measured cardiac parameters, CIMT, blood pressure, biomarkers or haemoglobin levels. Bone mineral density showed minimal changes in both arms and there was no significant difference.

### Post hoc analysis

When the trial population was considered as a whole, statistically significant reductions were seen for LVMI and CIMT (*P* = 0.03 and *P* = 0.001, respectively) between baseline and 12 months. This occurred despite progression of vascular calcification and stiffness. On multi-factorial analysis change of phosphate was significantly associated with change of CIMT (EE =0.007 (0.0, 0.13) *P* = 0.04). Change of LVMI was also associated with change of phosphate on uni-factorial (EE =0.95 (0.04, 1.86) *P* = 0.04) and multi-factorial analysis (EE =1.23 (0.01, 2.5) *P* = 0.05). In both cases improved phosphate control was associated with desirable reductions in the imaging parameter. Changes in FGF23 or iPTH showed no significant association with change in CIMT or LVMI.

Over the trial 22/36 (61%) patients had a reduction of phosphate (mean reduction of phosphate 0.3 mmol/L). There was no significant difference in starting dose or change of vitamin D doses administered between those who had a reduction in phosphate and those who did not.

During long term follow up there were 16 deaths from randomisation to censoring (median follow up of 32 months). There was no significant difference in the number of deaths between the groups (*P* = 0.74) (Table 4). In a univariable cox-regression model, cinacalcet status was not associated with all-cause mortality (HR:1.13; 95% CI: 0.39 to 3.2; *P* = 0.82) (Table 5).

**Table 4:** Outcomes on long term follow-up

Description	Cinacalcet arm (15)	Control arm (21)	*p*-Value
Follow up, months Median (IQR)	28.5 (22.2–47.5)	34.7 (15.5–72.8)	0.55*
Deaths, *n* (%)	6 (40)	10 (47.6)	0.74
Transplant, *n* (%)	5 (33.3)	5 (23.8)	1.0
Lost to follow-up, *n* (%)	4 (26.6)	5 (23.8)	1.0
Reached 31/12/2018, *n* (%)	0	1 (4.76)	1.0

*p*-Value by Fisher-exact test, *p- value by Mann-Whitney U test

**Table 5:** Association of intervention (cinacalcet use) with all-cause mortality (Cox-regression analysis- univariable model)

Variable	HR (95%CI)	p-Value
Cinacalcet	1.13 (0.39–3.2)	0.82
Age at baseline	1.07 (1.02–1.13)	0.013
Gender (Male)	2.71 (0.99–7.3)	0.05
Ethnicity (Caucasian)	2.3 (0.05–11.3)	0.47
Smoking at baseline	0.86 (0.29–2.5)	0.78

HR- Hazard ratio, CI- confidence interval

## Discussion

In this study we found no significant difference in progression of vascular calcification when cinacalcet was used to control secondary hyperparathyroidism alongside standard therapy compared to standard therapy alone when a policy of intensive management of CKD-MBD parameters was overseen. Cinacalcet has been shown to attenuate the progression of vascular calcification in rat models but human studies have shown a weaker effect [19, 20]. In the ADVANCE study there was a modest benefit with cinacalcet as opposed to standard therapy, the median percentage of coronary calcification progression being 24% in the cinacalcet arm vs. 31% in the control arm (*P* = 0.07). Although the primary endpoint was negative the difference reached significance when corrected for baseline phosphate.

Progression of vascular calcification in our study was comparable to that seen in other dialysis patient studies [12, 13]. The unrestricted use of vitamin D in our study, the tight control achieved for PTH and phosphate, the small sample size and low baseline calcification scores in some of the patients may account for the reduced progression of calcification between this and some other studies. The tight control, which was also achieved in the standard therapy arm, may have reduced the difference in effect between the two arms of the study. In the aggregated analysis of all patients change of coronary calcification was associated with higher baseline serum phosphate and FGF23 although this association was not shown with aortic calcification.

High levels of phosphate and iPTH have been shown to be associated with increased mortality and vascular calcification [21, 22]. In this study we reduced both phosphate and iPTH in both treatment arms by equal degrees. Patients in the ‘standard therapy’ arm of the RCT were encouraged to adhere to their mineral bone disease treatments (ie phosphate binders and vitamin D agonists) and most of them were compliant. This most likely explains how the serum phosphate and PTH were so well controlled in both groups at 12 months. With the exception of one patient in the control arm all patients included in the trial were receiving one-alphacalcidol. The dose of one-alphacalcidol varied widely between the patients (0.25 micrograms three times a week to four micrograms three times a week). None of the patients in the standard therapy (control) arm received cinacalcet during the course of the study.

There is probably a beneficial role for both cinacalcet and vitamin D analogues, however cinacalcet allows control of secondary hyperparathyroidism to be achieved more easily in patients [6]. The PARADIGM study was a randomised open label study to assess effect of cinacalcet versus vitamin D on biochemical parameters. This showed that both classes of therapy can reduce iPTH to a similar target but that their effects on calcium and phosphate concentrations differ [23]. There is data to suggest that cinacalcet may lower blood pressure, improve cardiac morphology and lower FGF23 and our study has also shown trends consistent with these studies [24–27]. St. Peter et al. found no relationship between short term change in iPTH due to cinacalcet use and cardiovascular outcomes although this was after only one year and so any beneficial changes may not have had time to take effect [28]. The relationship between cinacalcet and patient survival has been investigated in the largest study of CKD-MBD, the EVOLVE study [29]. The primary end-point of the trial was negative with intention-to-treat analysis but when baseline demographic variations were included in the analysis an improvement in survival was suggested [30]. In pre-specified secondary analyses older patients have been shown to have improved survival and reduced cardiovascular events if taking cinacalcet [31]. Although no difference in mortality was seen during long term follow up in our study, we recognise that the study was markedly underpowered to demonstrate this.

High iPTH levels have also been shown to be associated with left ventricular hypertrophy (LVH) in haemodialysis patients and the relationship between iPTH and LVH is thought to be independent of blood pressure [32, 33]. A reduction in LVMI was seen in both arms of our study with no significant difference between treatment arms. These data are interesting as this occurred despite progression of vascular stiffness and vascular calcification, and without changes in any other key parameters such as blood pressure or dialysis duration and suggests that tighter control of phosphate and/or iPTH may be associated with a reduction in LVMI. The trend to greater improvement observed in the cinacalcet arm may be explained by a number of mechanisms including the presence of the calcium sensing receptor (CaSR), identified on cardiac myocytes, which has been shown to affect DNA synthesis and may affect cell remodelling and growth [34, 35] An overall reduction in CIMT was also seen in this study when data in the two arms were aggregated.

We wished to further explore the regression of LVMI and CIMT and therefore performed post hoc analysis of the trial data, making use of an increased sample size and survival data. In these analyses we found a significant association between reduction in phosphate and regression of LVMI, reduction of CIMT and improved survival. These associations were not seen with iPTH reduction or change in FGF23 although this may be linked to the limitations of small patient numbers or short duration of the trial. There are many studies detailing that increased phosphate is associated with an increased mortality risk in CKD and dialysis patients [36, 37]. Higher serum phosphate is associated with LVH in the general population, predialysis and dialysis patients and it follows, but is not causally proven, that lowering phosphate may be beneficial [38–40]. There are many studies comparing different phosphate binders and morbidity but there is an evidence gap with respect to the reduction of elevated phosphate leading to improved patient outcomes [12, 13]. Our study has shown that patients having a reduction in phosphate had an improved survival over four years although the patient numbers were very small, and it is important to emphasise that this was a post-hoc analysis. Although interesting, a larger clinical trial would be needed to verify our findings.

Our overall findings support the view that tight control of both phosphate and iPTH, by whatever means, may slow progression of calcification and reduce LVMI and CIMT. However, the potential detrimental effects of the medications used to achieve targets need to be considered. There are studies suggesting increased calcification with high dose vitamin D and calcium containing phosphate binders [41, 42]. A proportion of patients within our study also developed low iPTH levels, despite close monitoring and dose adjustments, and therefore had increased likelihood of developing adynamic bone disorder. Both of these complications are associated with a worse overall outcome [43].

The main limitation of this study was the small sample size. In light of the ADVANCE study it is clear that a larger sample size would be required to show conclusive changes in vascular calcification. However, despite only 36 patients entering the study, the changes in calcification favoured cinacalcet use, reflecting the tight control of phosphate and iPTH achieved in our study, brought about by diligent and frequent monitoring by a clinician, which would be difficult to achieve in the setting of a larger trial. This could be achievable in clinical practice albeit at the expense of significant clinician resource. Although the post hoc analysis of our RCT generated interesting results regarding phosphate control, the study was not designed or powered to examine the effects of changes in phosphate level. However, our findings are hypothesis generating and further studies designed to confirm or refute the validity of these findings are indicated.

In conclusion, although this was a small RCT, it was very comprehensive in evaluating the effects of cinacalcet versus standard care on biochemical, cardiac, vascular and skeletal changes in haemodialysis patients with advanced hyperparathyroidism. Few if any other studies have examined the effects of cinacalcet, or indeed phosphate lowering, on cardiac MR imaging and the skeletal system as evidenced by bone densitometry. The most notable finding was that control of hyperparathyroidism and phosphate, irrespective of how this was achieved, was associated with a reduction in left ventricular mass at magnetic resonance.

## Supplementary Information

**Additional file 1: Appendix 2-** Targets for treatment (and startegy as to how to achieve the Targets).

**Additional file 2: Supplementary file 2.** Distribution of the weights generated by inverse probability of treatment weighting method using propensity scores (1-cinacalcet arm, 0-standard arm)

**Additional file 3: Supplementary file 3.** Estimated effect of the intervention (cinacalcet) on the difference in the outcome measures between baseline and follow-up in an inverse probability of treatment weighting method using propensity scores.

## Data Availability

The datasets used and/or analysed during the current study are available from the corresponding author on reasonable request.

## Abbreviations

Aix: Augmentation index
CIMT: Carotid intima-media thickness
CKD: Chronic kidney disease
CKD-MBD: Chronic kidney disease mineral and bone disorder
CMR: Cardiac magnetic resonance
EE: Estimated effect
IPTW: Inverse probability of treatment weighting
IQR: Interquartile range
LVH: Left ventricular hypertrophy
LVMI: Left ventricular mass index
MHRA: Medicines & Healthcare products Regulatory Agency
PTH: Parathormone
PWV: Pulse wave velocity
SD: Standard deviation
RCT: Randomised control trial
SHPT: Secondary hyperparathyroidism

## References

[1] Thompson S, James M, Wiebe N, Hemmelgarn B, Manns B, Klarenbach S (2015). Cause of death in patients with reduced kidney function. J Am Soc Nephrol.

[2] Ulusoy S, Ozkan G, Guvercin B, Yavuz A (2016). The relation between variability of intact parathyroid hormone, calcium, and cardiac mortality in hemodialysis patients. Artif Organs.

[3] Villa-Bellosta R, Rodriguez-Osorio L, Mas S, Abadi Y, Rubert M, De La Piedra C (2017). A decrease in intact parathyroid hormone (iPTH) levels is associated with higher mortality in prevalent hemodialysis patients. Jha V, editor. PLoS One.

[4] Wu GY, Da Xu B, Wu T, Wang XY, Wang TX, Zhang X (2016). Correlation between serum parathyroid hormone levels and coronary artery calcification in patients without renal failure. Biomed Rep.

[5] Brown SJ, Ruppe MD, Tabatabai LS (2017). The parathyroid gland and heart Disease. Methodist Debakey Cardiovasc J.

[6] Moe SM, Chertow GM, Coburn JW (2005). Achieving NKF-K/DOQI™ bone metabolism and disease treatment goals with cinacalcet HCl. Kidney Int.

[7] Pereira L, Meng C, Marques D, Frazão JM (2018). Old and new calcimimetics for treatment of secondary hyperparathyroidism: impact on biochemical and relevant clinical outcomes. Clin Kidney J.

[8] Jung S, Querfeld U, Müller D, Rudolph B, Peters H, Krämer S (2012). Submaximal suppression of parathyroid hormone ameliorates calcitriol-induced aortic calcification and remodeling and myocardial fibrosis in uremic rats. J Hypertens.

[9] Ivanovski O, Nikolov IG, Joki N, Caudrillier A, Phan O, Mentaverri R (2009). The calcimimetic R-568 retards uremia-enhanced vascular calcification and atherosclerosis in apolipoprotein E deficient (apoE−/−) mice. Atherosclerosis..

[10] Raggi P, Chertow GM, Torres PU, Csiky B, Naso A, Nossuli K (2011). The ADVANCE study: a randomized study to evaluate the effects of cinacalcet plus low-dose vitamin D on vascular calcification in patients on hemodialysis. Nephrol Dial Transplant.

[11] Liu L, Wang Y, Chen H, Zhu X, Zhou L, Yang Y (2014). The effects of non-calcium-based phosphate binders versus calcium-based phosphate binders on cardiovascular calcification and bone remodeling among dialysis patients: a meta-analysis of randomized trials. Ren Fail.

[12] Kakuta T, Tanaka R, Hyodo T, Suzuki H, Kanai G, Nagaoka M (2011). Effect of sevelamer and calcium-based phosphate binders on coronary artery calcification and accumulation of circulating advanced glycation end products in hemodialysis patients. Am J Kidney Dis.

[13] Qunibi W, Moustafa M, Muenz LR, He DY, Kessler PD, Diaz-Buxo JA (2008). A 1-year randomized trial of calcium acetate versus Sevelamer on progression of coronary artery calcification in hemodialysis patients with comparable lipid control: the calcium acetate Renagel Evaluation-2 (CARE-2) study. Am J Kidney Dis.

[14] Floege J, Raggi P, Block GA, Torres PU, Csiky B, Naso A (2010). Study design and subject baseline characteristics in the ADVANCE study: effects of cinacalcet on vascular calcification in haemodialysis patients. Nephrol Dial Transplant.

[15] Kidney Disease (2009). Improving Global Outcomes (KDIGO) CKD-MBD Work Group. KDIGO clinical practice guideline for the diagnosis, evaluation, prevention, and treatment of chronic kidney disease-mineral and bone disorder (CKD-MBD). Kidney Int.

[16] Floege J, Kim J, Ireland E, Chazot C, Drueke T, De Francisco A (2011). Serum iPTH, calcium and phosphate, and the risk of mortality in a European haemodialysis population. Nephrol Dial Transplant.

[17] Sakaguchi T, Akizawa T. K/DOQI clinical practice guidelines for bone metabolism and disease in CKD. Vol. 42. Am J Kidney Dis. 2003:S1–201. 14520607

[18] Raad H, Cornelius V, Chan S, Williamson E, Cro S (2020). An evaluation of inverse probability weighting using the propensity score for baseline covariate adjustment in smaller population randomised controlled trials with a continuous outcome. BMC Med Res Methodol.

[19] Henley C, Davis J, Miller G, Shatzen E, Cattley R, Li X (2009). The calcimimetic AMG 641 abrogates parathyroid hyperplasia, bone and vascular calcification abnormalities in uremic rats. Eur J Pharmacol.

[20] Lopez I, Mendoza FJ, Guerrero F, Almaden Y, Henley C, Aguilera-Tejero E (2009). The calcimimetic AMG 641 accelerates regression of extraosseous calcification in uremic rats. Am J Physiol Ren Physiol.

[21] Naves-Daz M, Passlick-Deetjen J, Guinsburg A, Marelli C, Fernndez-Martn JL, Rodrguez-Puyol D (2011). Calcium, phosphorus, PTH and death rates in a large sample of dialysis patients from Latin America. The CORES study. Nephrol Dial Transplant.

[22] Custódio MR, Koike MK, Neves KR, Dos Reis LM, Graciolli FG, Neves CL (2012). Parathyroid hormone and phosphorus overload in uremia: impact on cardiovascular system. Nephrol Dial Transplant.

[23] Wetmore JB, Gurevich K, Sprague S, Da Roza G, Buerkert J, Reiner M (2015). A randomized trial of cinacalcet versus vitamin D analogs as monotherapy in secondary hyperparathyroidism (PARADIGM). Clin J Am Soc Nephrol.

[24] Odenwald T, Nakagawa K, Hadtstein C, Roesch F, Gohlke P, Ritz E (2006). Acute blood pressure effects and chronic hypotensive action of calcimimetics in uremic rats. J Am Soc Nephrol.

[25] Ogata H, Ritz E, Odoni G, Amann K, Orth SR (2003). Beneficial effects of calcimimetics on progression of renal failure and cardiovascular risk factors. J Am Soc Nephrol.

[26] Wetmore JB, Liu S, Krebill R, Menard R, Quarles LD (2010). Effects of cinacalcet and concurrent low-dose vitamin D on FGF23 levels in ESRD. Clin J Am Soc Nephrol.

[27] Sprague SM, Wetmore JB, Gurevich K, Da Roza G, Buerkert J, Reiner M (2015). Effect of cinacalcet and vitamin D analogs on fibroblast growth factor-23 during the treatment of secondary hyperparathyroidism. Clin J Am Soc Nephrol.

[28] Peter WLS, Yusuf AA, Do T, Lowe KA, Liu J, Nieman KM (2015). Parathyroid hormone change after cinacalcet initiation and one-year clinical outcome risk: a retrospective cohort study. BMC Nephrol.

[29] Chertow GM, Pupim LB, Block GA, Correa-Rotter R, Drueke TB, Floege J (2007). Evaluation of Cinacalcet therapy to lower cardiovascular events (EVOLVE): rationale and design overview. Clin J Am Soc Nephrol.

[30] Block GA, Correa-Rotter R, Drüeke TB, Floege J, Goodman WG, Herzog CA (2012). Effect of cinacalcet on cardiovascular disease in patients undergoing dialysis. N Engl J Med.

[31] Parfrey PS, Drüeke TB, Block GA, Correa-Rotter R, Floege J, Herzog CA, et al. Evaluation of Cinacalcet HCl Therapy to Lower Cardiovascular Events (EVOLVE) Trial Investigators. The effects of cinacalcet in older and younger patients on hemodialysis: the evaluation of cinacalcet HCL therapy to lower cardiovascular events (EVOLVE) trial. Clin J Am Soc Nephrol 2015;10(5):791–799, DOI: 10.2215/CJN.07730814. 10.2215/CJN.07730814 PMC4422239 25710802

[32] Al-Hilali N, Hussain N, Ataia A, Al-Azmi M, Al-Helal B, Johny K (2009). Hypertension and hyperparathyroidism are associated with left ventricular hypertrophy in patients on hemodialysis. Indian J Nephrol.

[33] Randon RB, Rohde LE, Comerlato L, Ribeiro JP, Manfro RC (2005). The role of secondary hyperparathyroidism in left ventricular hypertrophy of patients under chronic hemodialysis. Braz J Med Biol Res.

[34] Karohl C, Raggi P (2012). Cinacalcet: will it play a role in reducing cardiovascular events?. Futur Cardiol.

[35] Tfelt-Hansen J, Hansen JL, Smajilovic S, Terwilliger EF, Haunso S, Sheikh SP (2006). Calcium receptor is functionally expressed in rat neonatal ventricular cardiomyocytes. Am J Physiol Heart Circ Physiol.

[36] Hou Y, Li X, Sun L, Qu Z, Jiang L, Du Y. Phosphorus and mortality risk in end-stage renal disease: A meta-analysis. Vol. 474. Clin Chim Acta Elsevier B.V. 2017:108–13. 10.1016/j.cca.2017.09.005 28903022

[37] Palmer SC, Hayen A, Macaskill P, Pellegrini F, Craig JC, Elder GJ, et al. Serum levels of phosphorus, parathyroid hormone, and calcium and risks of death and cardiovascular disease in individuals with chronic kidney disease a systematic review and meta-analysis. Vol. 305. JAMA. 2011:1119–27. 10.1001/jama.2011.308 21406649

[38] Foley RN, Collins AJ, Herzog CA, Ishani A, Kalra PA (2009). Serum phosphate and left ventricular hypertrophy in young adults: the coronary artery risk development in young adults study. Kidney Blood Press Res.

[39] Chue CD, Edwards NC, Moody WE, Steeds RP, Townend JN, Ferro CJ (2012). Serum phosphate is associated with left ventricular mass in patients with chronic kidney disease: a cardiac magnetic resonance study. Heart..

[40] Patel RK, Oliver S, Mark PB, Powell JR, McQuarrie EP, Traynor JP (2009). Determinants of left ventricular mass and hypertrophy in hemodialysis patients assessed by cardiac magnetic resonance imaging. Clin J Am Soc Nephrol.

[41] Cardús A, Panizo S, Parisi E, Fernandez E, Valdivielso JM (2007). Differential effects of vitamin D analogs on vascular calcification. J Bone Miner Res.

[42] Lomashvili KA, Wang X, O’Neill WC (2014). Role of local versus systemic vitamin D receptors in vascular calcification. Arterioscler Thromb Vasc Biol.

[43] Bover J, Ureña P, Brandenburg V, Goldsmith D, Ruiz C, DaSilva I, et al. Adynamic Bone Disease: From Bone to Vessels in Chronic Kidney Disease. Vol. 34. Semin Nephrol. 2014:626–40. 10.1016/j.semnephrol.2014.09.008 25498381

