# Supplementary material for: A randomised controlled trial to examine the effects of cinacalcet on bone and cardiovascular parameters in haemodialysis patients with advanced secondary hyperparathyroidism
Source: BMC Nephrol. 2021 Mar 23;22:106. doi: 10.1186/s12882-021-02312-2 (PMC7989372; doi:10.1186/s12882-021-02312-2)
Supplement: Supplementary file 1 — Additional file 1: Appendix 2- Targets for treatment (and startegy as to how to achieve the Targets). [file 12882_2021_2312_MOESM1_ESM.pdf]

Authors: Helen Eddington PhD, Rajkumar Chinnadurai PhD, Helen Alderson PhD, Sara T Ibrahim MSc, Constantina Chrysochou PhD, Darren Green PhD, Ibi Erekosima MBBS, Alastair Hutchison, FRCP, MD, Abdalla Bubtana MBBS, Janet Hegarty MB ChB, Philip A Kalra FRCP, MD

Appendix 2- TARGETS FOR TREATMENT (AND STRATEGY AS TO HOW TO ACHIEVE THE TARGETS)

Phosphate:	<1.8mmol/L	(renal association 2002)
Corrected calcium:	2.2-2.6mmol/L	(renal association 2002)
PTH:	150-300pg /ml	(K/DOQI 2004)

These targets will be aimed for using the following algorithm

To go through algorithm you will need corrected calcium, phosphate and PTH. Use PTH to find appropriate chart then follow instructions according to calcium and phosphate result.

If PTH has not been measured use most recent PTH.

Ensure PTH measured at least 3 monthly during maintenance phase.

PTH < 150pg/ml

Pts on cinacalcet and std therapy	Pts on std therapy
**Ca <2.2 PO4 <=1.8**	**Ca <2.2 PO4 <=1.8**
Decrease dose of cinacalcet 1 step<br>If no change after 1-2 week or if calcium <1.9 add/increase calcium supplementation 1 step.	Add/increase calcium supplementation 1 step<br>If no change after 1-2 week or if calcium <1.9 then increase dose of Vitamin D 1 step
**Ca <2.2 PO4 >1.8**	**Ca <2.2 PO4 >1.8**
Increase dose of ca containing phosphate binders 1 step	Increase dose of ca containing phosphate binders 1 step
**Ca >2.6 PO4 <=1.8**	**Ca >2.6 PO4 <=1.8**
Decrease dose of ca containing phosphate binders 1 step<br>If no improvement in 1-2 week or not on ca phosphate binders then decrease dose of Vitamin D 1 step	Decrease dose of ca containing phosphate binders 1 step<br>If no improvement in 1-2 week or not on ca phosphate binders then decrease dose of Vitamin D 1 step
**Ca >2.6 PO4 >1.8**	**Ca high PO4 high**
Decrease Vitamin D 1 step<br>If not on vit D then increase dose of non-ca containing phosphate binders 1 step	Decrease Vitamin D 1 step<br>If not on vit D then increase dose of non-ca containing phosphate binders
**Ca 2.2-2.6 PO4 <0.6**	**Ca 2.2-2.6 PO4 <0.6**
Encourage dietary intake<br>And reduce dose of cinacalcet 1 step	Encourage dietary intake
**Ca 2.2-2.6 PO4 >1.8**	**Ca 2.2-2.6 PO4 >1.8**
Increase dose of non-ca containing phosphate binders 1 step	Increase dose of non-ca containing phosphate binders 1 step
**Ca 2.2-2.6 PO4 <1.8**	**Ca 2.2-2.6 PO4 <1.8**
Decrease vitamin D 1 step<br>If PTH not between 150-300 next checked then decrease dose of cinacalcet 1 step	Decrease Vitamin D

PTH 150-300pg/ml

**Pts on cinacalcet and std therapy**	**Pts on std therapy**
Ca <2.2 PO4 <=1.8	Ca <2.2 PO4 <=1.8
Increase dose of Vitamin D 1 step<br>If no improvement in 1-2 weeks then add calcium supplementation 1 step	Increase dose of Vitamin D 1 step<br>If no improvement after 1-2 weeks then add calcium supplementation 1 step
Ca <2.2 PO4 >1.8	Ca <2.2 PO4 >1.8
Increase ca containing phosphate binders 1 step	Increase ca containing phosphate binders 1 step
Ca >2.6 PO4 <=1.8	Ca >2.6 PO4 <=1.8
Decrease ca containing phosphate binders 1 step<br>If not on, then decrease vit D 1 step,<br>If not on then increase cinacalcet 1 step	Decrease ca containing phosphate binders 1 step<br>If not on, decrease vit D 1 step
Ca >2.6 PO4 >1.8	Ca >2.6 PO4 >1.8
Decrease Vitamin D 1 step<br>If not on, decrease ca containing phosphate binders and increase non calcium containing phosphate binders 1 step each<br>If not on vit D or calcium phosphate binders then increase dose of cinacalcet 1 step	Decrease Vitamin D 1 step<br>If not on, decrease ca containing phosphate binders and increase non calcium containing phosphate binders 1 step each
Ca 2.2-2.6 PO4 <0.6	Ca 2.2-2.6 PO4 <0.6
Encourage dietary intake	Encourage dietary intake
Ca 2.2-2.6 PO4 >1.8	Ca 2.2-2.6 PO4 >1.8
Increase dose of non-ca containing phosphate binders 1 step<br>If no change after 1-2 weeks increase dose on cinacalcet 1 step	Increase dose of non-ca containing phosphate binders 1 step
Ca 2.2-2.6 PO4 <=1.8	Ca 2.2-2.6 PO4 <=1.8
No change	No change

PTH >300 pg/ml

Pts on cinacalcet and std therapy	Pts on std therapy
Ca <2.2 PO4 <=1.8	Ca low PO4 =/low
Increase Vitamin D 1 step	Increase Vitamin D 1 step
Ca <2.2 PO4 >1.8	Ca <2.2 PO4 >1.8
Increase ca containing phosphate binders 1 step	Increase ca containing phosphate binders 1 step
Ca >2.6 PO4 <=1.8	Ca >2.6 PO4 <=1.8
Increase cinacalcet 1 step<br>And decrease calcium phosphate binders 1 step	Decrease calcium containing phosphate binders 1 step<br>If not on ca phosphate binders then decrease vitamin D 1 step
Ca >2.6 PO4 >1.8	Ca >2.6 PO4 >1.8
Increase cinacalcet 1 step<br>If on maximum dose then decrease vitamin D<br>If on maximum cinacalcet and not on vit D then increase non-ca containing phosphate binders 1 step and decrease ca phosphate binders 1 step	Decrease vitamin D 1 step and increase non-ca containing phosphate binders 1 step<br>If not on vit D then increase non-ca containing phosphate binders 1 step and decrease ca phosphate binders 1 step
Ca 2.2-2.6 PO4 <1.8	Ca 2.2-2.6 PO4 <1.8
Increase cinacalcet 1 step<br>If on maximum dose increase vitamin D 1 step	Increase vitamin D 1 step
Ca 2.2-2.6 PO4 >1.8	Ca 2.2-2.6 PO4 >1.8
Increase cinacalcet 1 step<br>If on maximum dose then increase non-ca containing phosphate binders 1 step	Increase non-ca containing phosphate binders 1 step
Ca 2.2-2.6 PO4 <1.8	Ca 2.2-2.6 PO4 <1.8
Increase cinacalcet 1 step<br>If on maximum dose increase vitamin D 1 step	Increase vitamin D 1 step

Cinacalcet:

1. 30mg
2. 60mg
3. 90mg
4. 120mg
5. 150mg
6. 180mg max dose

Calcium supplementation / phosphate binders:

Use calcium containing compound

Calcichew = 500mg calcium
Calcium acetate(phosex) = 250mg
Calcium 500 = 500mg

Total daily dose increments:

1. 500mg
2. 1000mg
3. 1500mg
4. 2000mg
5. 2500mg
6. 3000mg
7. 4000mg

Patients may need to miss a dose increment depending on which tablet they are taking. This is because not all patients are tolerant of all types mentioned.

Non-calcium containing phosphate binders

Sevelamer 800mg

Total daily dose increments:

1. 1600mg
2. 2400mg
3. 4000mg
4. 4800mg
5. 6400mg
6. 7200mg

Patients may jump an increment if felt clinically necessary. No maximum dose, if higher doses required please discuss with one of clinicians involved in the trial.

Vitamin D

Available compounds:
One-alpha available in 0.25mcg, 0.5mcg and 1 mcg tablets
Calcitriol 0.25mcg, 0.5 mcg

0.25 micrograms (mcg) = 250 nanograms (ng)

Tablets can be given daily, twice a week or three times a week

Total **WEEKLY** dose increments:

1. 0.5mcg
2. 1.0 mcg
3. 1.5 mcg
4. 2.0 mcg
5. 2.5mcg
6. 3.0 mcg
7. 3.5mcg
8. 4.0 mcg
9. 5.0mcg
10. 6.0mcg
11. 7.0mcg

Maximum dose is 35mcg per week. If further increases are necessary then consult clinician involved in trial.
Patients may jump an increment if felt necessary by clinician
