# Supplementary material for: A randomised controlled trial to examine the effects of cinacalcet on bone and cardiovascular parameters in haemodialysis patients with advanced secondary hyperparathyroidism
Source: BMC Nephrol. 2021 Mar 23;22:106. doi: 10.1186/s12882-021-02312-2 (PMC7989372; doi:10.1186/s12882-021-02312-2)

**A randomised controlled trial to examine the effects of cinacalcet on bone and cardiovascular parameters in haemodialysis patients with advanced**

**secondary hyperparathyroidism**

**Authors:** Helen Eddington PhD, Rajkumar Chinnadurai PhD, Helen Alderson PhD, Sara T Ibrahim MSc, Constantina Chrysochou PhD, Darren Green PhD, Ibi Erekosima MBBS, Alastair Hutchison, FRCP, MD, Abdalla Bubtana MBBS, Janet Hegarty MB ChB, Philip A Kalra FRCP, MD

**Supplementary file 2. Distribution of the weights generated by inverse probability of treatment weighting method using propensity scores (1-cinacalcet arm, 0-standard arm)**


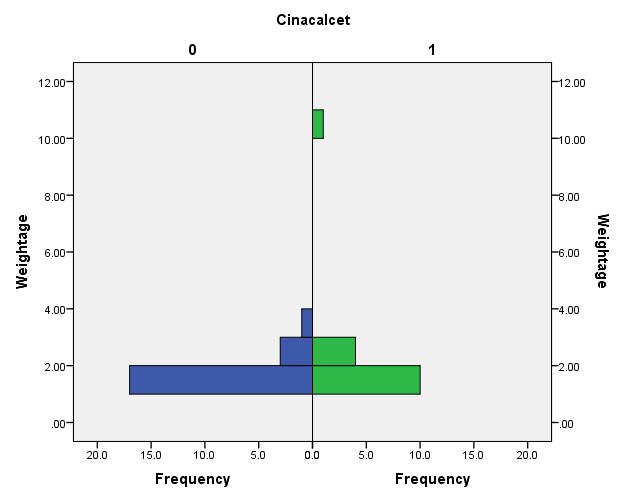

Supplement: Supplementary file 2 — Additional file 2: Supplementary file 2. Distribution of the weights generated by inverse probability of treatment weighting method using propensity scores (1-cinacalcet arm, 0-standard arm) [file 12882_2021_2312_MOESM2_ESM.docx]
