# Supplementary material for: A randomised controlled trial to examine the effects of cinacalcet on bone and cardiovascular parameters in haemodialysis patients with advanced secondary hyperparathyroidism
Source: BMC Nephrol. 2021 Mar 23;22:106. doi: 10.1186/s12882-021-02312-2 (PMC7989372; doi:10.1186/s12882-021-02312-2)
Supplement: Supplementary file 3 — Additional file 3: Supplementary file 3. Estimated effect of the intervention (cinacalcet) on the difference in the outcome measures between baseline and follow-up in an inverse probability of treatment weighting method using propensity scores. [file 12882_2021_2312_MOESM3_ESM.docx]

**A randomised controlled trial to examine the effects of cinacalcet on bone and cardiovascular parameters in haemodialysis patients with advanced**

**secondary hyperparathyroidism**

**Authors:** Helen Eddington PhD, Rajkumar Chinnadurai PhD, Helen Alderson PhD, Sara T Ibrahim MSc, Constantina Chrysochou PhD, Darren Green PhD, Ibi Erekosima MBBS, Alastair Hutchison, FRCP, MD, Abdalla Bubtana MBBS, Janet Hegarty MB ChB, Philip A Kalra FRCP, MD

**Supplementary file 3. Estimated effect of the intervention (cinacalcet) on the difference in the outcome measures between baseline and follow-up in an inverse probability of treatment weighting method using propensity scores.**

|  | Estimated effect (95% CI) | p-Value |
| --- | --- | --- |
| Coronary calc score | -172.2 (-554.1 to 209.62) | 0.337 |
| Total calc score | 59.9 (-1052.34 to 1172.17) | 0.497 |
| LV mass index (g/m^2^) | -27.3 (-56.8 to 2.32) | 0.071 |
| CIMT (mm) | -0.004 (-0.010 to 0.002) | 0.181 |
| cfPWV (m/s) | 0.28 (-1.05 to 1.61) | 0.679 |

cfPWV: carotid-femoral pulse wave velocity: CIMT: carotid intima-media thickness; calc: calcification; LV: left ventricular
